# Unveiling the Role of Fluorination in Suppressing Dark Current and Enhancing Photocurrent to Enable Thick-Film Near-Infrared Organic Photodetectors

**DOI:** 10.3390/polym17192663

**Published:** 2025-10-01

**Authors:** Yongqi Bai, Seon Lee Kwak, Jong-Woon Ha, Do-Hoon Hwang

**Affiliations:** 1 Department of Chemistry, Pusan National University, 2 Busandaehak-ro, Geumjeong-gu, Busan 46241, Republic of Korea; byongqi@163.com (Y.B.); gsun0331@naver.com (S.L.K.); 2 Department of Materials Engineering and Convergence Technology, Gyeongsang National University, Jinju 52828, Republic of Korea

**Keywords:** organic photodetectors, thick photoactive layer, high charge mobility, near-infrared, low dark current

## Abstract

Thick active layers are crucial for scalable production of organic photodetectors (OPDs). However, most OPDs with active layers thicker than 200 nm typically exhibit decreased photocurrents and responsivities due to exciton diffusion and prolonged charge transport pathways. To address these limitations, we designed and synthesized PFBDT-8ttTPD, a fluorinated polymer donor. The strategic incorporation of fluorine effectively enhanced the charge carrier mobility, enabling more efficient charge transport, even in thicker films. OPDs combining PFBDT−8ttTPD with IT−4F or Y6 non-fullerene acceptors showed a substantially lower dark current density (*J*_d_) for active layer thicknesses of 250−450 nm. Notably, *J*_d_ in the IT-4F-based devices declined from 8.74 × 10^−9^ to 4.08 × 10^−10^ A cm^−2^ under a reverse bias of −2 V, resulting in a maximum specific detectivity of 3.78 × 10^13^ Jones. Meanwhile, Y6 integration provided near-infrared sensitivity, with the devices achieving responsivity above 0.48 A W^−1^ at 850 nm and detectivity over 10^13^ Jones up to 900 nm, supporting broadband imaging. Importantly, high-quality thick films (≥400 nm) free of pinholes or defects were fabricated, enabling scalable production without performance loss. This advancement ensures robust photodetection in thick uniform layers and marks a significant step toward the development of industrially viable OPDs.

## 1. Introduction

In recent years, organic photodetectors (OPDs) have gained significant attention owing to their intrinsic advantages including flexibility, low−cost production via solution processing, controllable absorption wavelength, and higher intrinsic absorption coefficient compared with Si-based photodetectors [1,2,3,4,5,6]. The ability to tune the band gap of organic materials effectively is crucial for expanding the detection range to the near-infrared (NIR) region. This expansion will allow OPDs to be applied to diverse optoelectronic devices including image sensors, fingerprint scanners, biomedical sensors, and machine vision [7,8,9,10]. Recent development of non-fullerene acceptors (NFAs) has significantly extended the NIR photoresponse beyond 900 nm [11,12,13,14,15]. Yet, it remains very challenging to simultaneously achieve high detectivity (D*) and low dark current density (*J*_d_) at these wavelengths, particularly when using scalable processes such as slot-die and roll-to-roll printing that often produce thick or non-uniform active layers.

Organic semiconductors typically possess short exciton diffusion lengths (generally <10 nm), requiring active layers thinner than 100 nm for effective exciton dissociation. However, practical printing processes often demand substantially thicker films to prevent pinholes and enhance uniformity [16,17,18]. As a result, films thicker than 200 nm exhibit considerable photocurrent losses in NFA-based OPDs due to low electron mobility of NFAs, which promotes recombination of excitons generated deep within the films [19]. Furthermore, while thicker films can suppress pinhole defects and ensure uniformity in practical printing, they also extend the charge−transport pathway and aggravate surface inhomogeneity, further degrading the photocurrent and D* [20,21,22,23]. OPDs based on fullerene derivatives have higher electron mobilities (typically ~10^−3^ cm^2^ V^−1^ s^−1^) and therefore can partially mitigate these losses. For instance, Zeng et al. [24]. reported a 400 nm-thick PNTT:PC_71_BM device with a *J*_d_ of 0.33 nA cm^−2^ under a reverse bias of –0.1 V and a specific D* of 4 × 10^13^ Jones at 760 nm. Given that the absorption of PCBM sharply declines beyond ~550 nm and becomes negligible above ~700 nm, the response of the PNTT:PC_71_BM device at 760 nm relies exclusively on the donor polymer’s absorption in the red region, highlighting the inherent limitations of fullerene acceptors for NIR detection [24]. Recent advances in NFAs have enabled OPDs with high photoresponse in the 800–1050 nm region [25,26,27,28]. Nevertheless, the intrinsically low electron mobilities of most NFAs lead to a rapid decline in photocurrent when the active layer is thicker than 200 nm. This is because excitons generated deep within the film recombine before extraction at the interface, owing to their limited diffusion and dissociation lengths [21,29]. Thus, the development of NFA-based devices that combine thick active layers (≥250 nm), high photocurrents, and low *J*_d_ remains an open and crucial challenge.

Owing to their high charge mobilities, thienopyrrolodione (TPD) −based polymer donors, notably PBDT−8ttTPD, have demonstrated the ability to sustain high D* with 1 μm-thick films when blended with PCBM [30]. In this study, we applied strategic fluorination to the backbone of PBDT−8ttTPD to address the thickness-related performance loss in NFA-based OPDs. The rationale for this synthetic strategy is twofold. First, strong fluorine–fluorine (F−F) interactions promote coherent π–π stacking and improve the film uniformity. This leads to a reduction in energetic disorder and the suppression of both shallow and deep trap-state densities, as evidenced by prior research [31]. Secondly, the high electronegativity of fluorine effectively stabilizes frontier molecular orbitals of the polymer. This results in a downward shift of both the highest occupied molecular orbital (HOMO) and lowest unoccupied molecular orbital (LUMO) energy levels, importantly, without altering the overall bandgap [31]. This deeper energy alignment subsequently reduces the charge−injection rates, thereby minimizing the dark hole injection. Furthermore, the strong interactions between F atoms in adjacent molecules also contribute to tighter molecular packing within the film, which is directly correlated with a higher charge-carrier mobility. Collectively, these synergistic effects are expected to enable the fabrication of thick-film (~450 nm) NFA-based OPDs that minimize *J*_d_ while simultaneously achieving efficient photocurrent extraction.

## 2. Materials and Methods

### 2.1. Measurements

UV-Vis spectra were measured using a JASCO JP/V-570 instrument (Tokyo, Japan). The dark current and photocurrent curves of the OPDs were obtained using a Keithley 2635 B System SourceMeter (Tektronix, Solon, OH, USA). The devices were measured under an NIR LED light (Thorlabs M810L4) (tHORLABS, Dachau, Germany) in air. The light intensity was adjusted using a standard photodiode power sensor (Thorlabs S120C). The external quantum efficiency (EQE) curves were measured using a McScience K3100 EQX system (Yeonggi-do, Republic of Korea). The EQE data were recorded as a function of wavelength in the range of 300-1100 nm using a xenon short-arc lamp as the light source, and calibration was performed using a Si photodiode. Grazing incident wide-angle X-ray scattering (GIWAXS) measurements were performed at beamline 3C in Pohang Accelerator Laboratory (Pohang in Republic of Korea).

### 2.2. Materials

All reagents for polymer synthesis and device fabrication were purchased from Alfa Aesar (Ward Hill, MA, USA), Aldrich (St. Louis, MI, USA), or TCI Korea (Seoul, Republic of Korea). IT-4F and Y6 were purchased from 1-Material. MoO_3_ and Ag were purchased from iTASCO (Incheon, Republic of Korea).

### 2.3. Fabrication of OPDs

The OPDs were prepared with an inverted structure of indium tin oxide (ITO)/ZnO/active layer/MoO_3_/Ag. The ZnO solution was prepared as reported previously [32]. Active layer solutions at various concentrations (20 to 40 mg mL^−1^, polymer:NFAs = 1:1 *w*/*w*) were prepared using chloroform as the solvent and stirred overnight at 35 °C. The ITO substrates were sequentially cleaned in a hyperacoustic bath using detergent solution, distilled water, acetone, and then isopropanol for 10 min each. After treating the cleaned ITO substrates with UV-ozone plasma for 20 min, a ZnO layer was deposited on them according to the literature [32]. The active layer was coated in a glove box, and its thickness was controlled by changing the rotational speed and solution concentration. After drying the active layer, MoO_3_ and Ag were thermally evaporated under a base pressure of 10^−7^ torr. The effective area of the active layer was 0.3 cm × 0.3 cm.

## 3. Results

**Figure 1:**
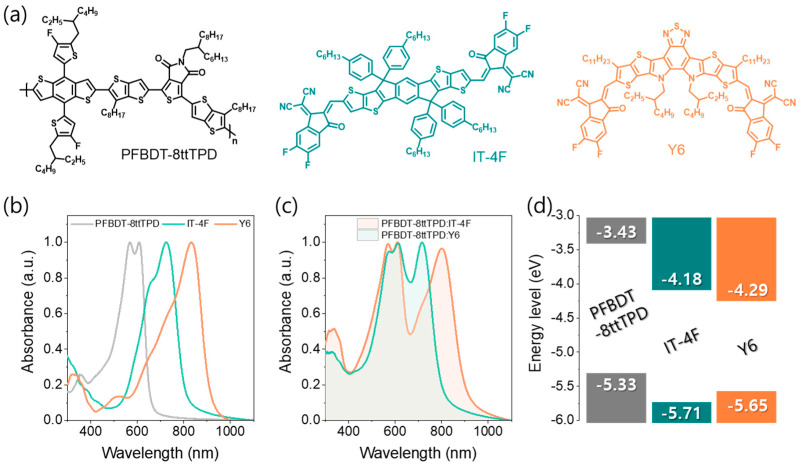
(**a**) Chemical structures of PFBDT−8ttTPD, IT−4F, and Y6. (**b**) Normalized UV−Vis absorption spectra of neat PFBDT−8ttTPD, IT−4F, and Y6 films. (**c**) Normalized UV−Vis absorption spectra of blend films. (**d**) Energy-level diagram of PFBDT−8ttTPD, IT−4F, and Y6 as determined by cyclic voltammetry.

Chemical structures of the donor polymer (PFBDT−8ttTPD) and two NFAs (IT−4F and Y6) are shown in Figure 1a. PFBDT−8ttTPD was synthesized via a Stille polycondensation reaction using Pd(PPh_3_)_4_ as the catalyst (Scheme S1). The resulting polymer exhibits excellent solubility (>20 mg mL^−1^) in common chlorinated solvents such as chloroform, chlorobenzene, and *o*-dichlorobenzene. As shown in Figure 1b, the UV-Vis absorption spectra of neat PFBDT−8ttTPD films reveal an absorption onset at app-roximately 650 nm, corresponding to an optical bandgap of approximately 1.9 eV. This absorption profile effectively complements those of both IT−4F and Y6. Notably, the PFBDT-8ttTPD:Y6 blend exhibited extended absorption into the NIR region, reaching approximately 900 nm, whereas the absorption of the PFBDT−8ttTPD:IT−4F blend was limited to approximately 800 nm (Figure 1c). These findings suggest that the PFBDT-8ttTPD:Y6 system holds significant promise for achieving photodetection in the high NIR range. Cyclic voltammetry was employed to determine the energy levels of all photoactive materials. Figure 1d shows the resulting cascade energy level alignment between PFBDT-8ttTPD and both NFAs, indicating efficient pathways for charge separation and collection.

**Figure 2:**
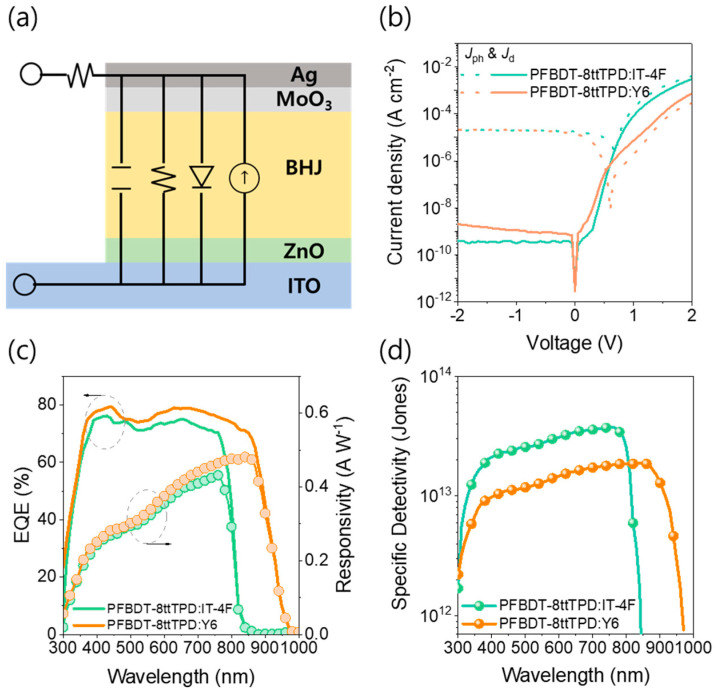
(**a**) Schematic illustration of the inverted configuration for OPDs. (**b**) Current−voltage characteristics measured in the dark and under standard solar illumination for PFBDT−8ttTPD:IT−4F (thickness: 450 nm) and PFBDT−8ttTPD:Y6 (420 nm). (**c**) External quantum efficiency and spectral responsivity curves for PFBDT−8ttTPD:IT−4F (450 nm) and PFBDT−8ttTPD:Y6 (420 nm). (**d**) Wavelength−dependent specific detectivity for PFBDT−8ttTPD:IT−4F (450 nm) and PFBDT−8ttTPD:Y6 (420 nm).

Inverted OPDs utilizing PFBDT−8ttTPD as the polymer donor were systematically evaluated for their photodetection performance. These OPDs have an architecture of ITO/ZnO/active layer/MoO_3_/Ag (as shown in Figure 2a), employing a donor:acceptor ratio of 1:1 (*w*/*w*) without additives and pre−annealed for 10 min at 90 °C. The active layer thickness was varied from 200 to 450 nm to investigate its impact on the device metrics; the details are summarized in Tables S1 and S2. The optimal device performance was observed at 450 nm for PFBDT-8ttTPD:IT−4F and 420 nm for PFBDT−8ttTPD:Y6. When the thickness increased to these optimal values, there was a notable reduction in *J*_d_ under a reverse bias of −2 V. For the IT−4F devices, *J*_d_ decreased significantly from 8.74 × 10^−9^ at 250 nm to 4.08 × 10^−10^ A cm^−2^ at 450 nm. In contrast, the Y6 devices reached a plateau in *J*_d_ at approximately 2.03 × 10^−9^ A cm^−2^ beyond a thickness of 420 nm. A substantial reduction in the *J*_d_ of OPDs was achieved by increasing the photoactive layer thickness [32,33]. Furthermore, the Y6-based OPDs exhibited a slightly higher *J*_d_ than their IT−4F counterparts. This difference is likely due to the lower effective bandgap of Y6 and the potentially higher hole-injection rate from the MoO_3_/Ag interface into Y6. Despite the inherently weaker internal electric field in thicker films, which could potentially suppress the photocurrent density (*J*_ph_), the measured *J*_ph_ remained high for all thicknesses, proving the compensatory role of the fluorinated polymer donor with excellent charge mobility. Indeed, the high charge carrier mobilities of PFBDT−8ttTPD (μₕ ≈ 3.3 × 10^−4^ cm^2^ V^−1^ s^−1^ and μₑ ≈ 2.3 × 10^−4^ cm^2^ V^−1^ s^−1^ [31]) are pivotal in sustaining efficient photocurrent extraction across the investigated thickness range (Figure 2b). The EQE spectra under −2 V bias were measured to determine the spectral responsivity (R = EQE × λ/1240). The PFBDT-8ttTPD:Y6 devices consistently outperformed the IT-4F devices in the visible-to-NIR spectrum (Figure 2c). The maximum *R* values were 0.43 A W^–1^ at 760 nm for PFBDT−8ttTPD:IT-4F and 0.48 A W^–1^ at 850 nm for PFBDT−8ttTPD:Y6. Remarkably, the PFBDT−8ttTPD:Y6 devices maintained a responsivity greater than 0.33 A W^–1^ even at 900 nm, highlighting their superior NIR photodetection performance. The specific D*, which was estimated using the equation D* = *R*/(2*qJ*_d_)^1/2^, reached an impressive value of 3.78 × 10^13^ Jones at 780 nm for PFBDT-8ttTPD:IT−4F. As shown in Figure 2d, the Y6−based OPDs also demonstrated competitive specific D* exceeding 1.89 × 10^13^ Jones at 850 nm and 1.29 × 10^13^ Jones at 900 nm, further confirming sensitivity in the NIR region (Table 1). Although the Y6−based OPDs exhibited a relatively higher *J*_d_ (approximately ×10^−9^ A cm^–2^) compared to PFBDT−8ttTPD:IT−4F, their overall performance remained competitive. Notably, these OPDs demonstrated effective photoresponse in the NIR region, underscoring their potentials for specific NIR applications. As presented in Table S1, our NFA-based OPDs deliver outstanding performance even at the thick photoactive layer, outperforming most reported NIR OPDs.

**Figure 3:**
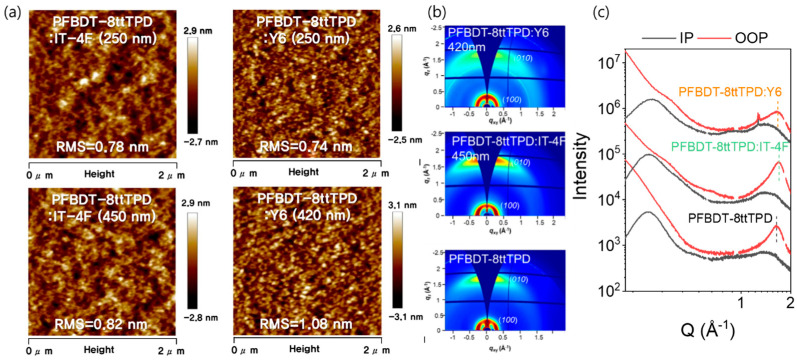
(**a**) AFM images of PFBDT-8ttTPD:NFAs at different active layer thicknesses. (**b**) 2D−GIWAXS images and (**c**) corresponding line−cut profile for neat PFBDT−8ttTPD, PFBDT−8ttTPD:IT−4F (450 nm), and PFBDT−8ttTPD:Y6 (420 nm).

Figure 3a shows the atomic force microscopy (AFM) topographies of the PFBDT−8ttTPD:IT−4F and PFBDT−8ttTPD:Y6 blend films with different thicknesses. Both systems maintained exceptionally low root−mean−square (RMS) roughness values (<1.1 nm) even as the film thickness increased. Specifically, the RMS roughness for the IT−4F blend increased from 0.78 to 0.82 nm, while for the Y6 blend it rose from 0.74 to 1.02 nm as the film thickness increased from 250 to 420 nm. Despite these slight increases in the RMS roughness, the film surfaces stayed exceptionally smooth and devoid of pinholes or defects, which is crucial for minimizing *J*_d_ in thick−film OPDs. Figure 3b,c present the two−dimensional grazing incidence wide−angle X−ray diffraction (2D-GIWAXS) images and the corresponding line−cut profiles along the in−plane (IP) and out−of−plane (OOP) directions. Neat PFBDT-8ttTPD exhibits lamellar stacking at q ≈ 0.28 Å^−1^ (d ≈ 22.4 Å and crystal coherence length (*L*_c_) ≈ 78.5 Å) and π−π stacking at q ≈ 1.64 Å^−1^ (d ≈ 3.83 Å and *L*_c_ ≈ 18.5 Å), consistent with a dominant face-on orientation. Upon blending with IT−4F, the π−π stacking distance contracts to ~3.63 Å, and *L*_c_ increases to ~31.4 Å, reflecting tighter molecular packing and larger crystalline domains. In contrast, the Y6 blend maintains a π−π stacking distance (~3.78 Å) similar to that of neat PFBDT−8ttTPD, but shows a reduced coherence length (~18.0 Å), indicative of smaller domain sizes. Based on the GIWAXS analysis, the PFBDT−8ttTPD:IT−4F blend exhibits a shorter π−π stacking distance and a substantially longer coherence length, suggesting the preservation of long−range molecular order even in thick films. This dense crystalline packing shortens the vertical charge−transport pathways and suppresses trap density, thereby enabling the simultaneous minimization of *J*_d_ and enhancement of *J*_ph_ in PFBDT−8ttTPD:IT−4F. The PFBDT−8ttTPD:Y6 blend maintains a comparable π−π spacing but displays a shorter *L*_c_, indicating smaller crystalline domains. Although this may increase carrier recombination at the domain boundaries, the Y6-based films capitalizes on their intrinsic NIR absorption to sustain photocurrent response up to 900 nm, demonstrating exceptional deep−NIR sensitivity. Ultimately, these GIWAXS−derived insights into the molecular ordering and domain size help explain how low *J*_d_ and high D* can be simultaneously achieved in thick-film NFA−based OPDs.

**Figure 4:**
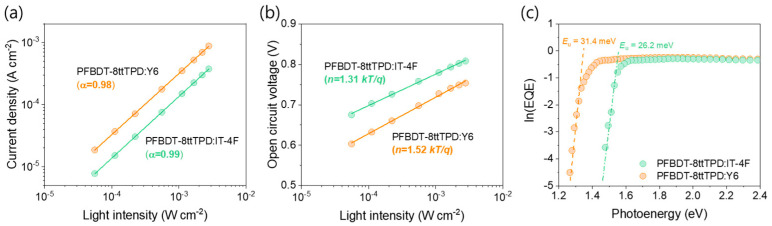
Variation in (**a**) short−circuit current density and (**b**) open−circuit voltage as a function of light intensity for PFBDT-8ttTPD:NFAs. (**c**) Extraction of Urbach energy from ln(EQE) in the long-wavelength edge for PFBDT-8ttTPD:NFAs.

The current densities of the PFBDT−8ttTPD-based OPDs were measured as a function of LED illumination intensity (*I_a_*) in order to probe both bimolecular and trap-assisted recombinations in these devices (Figure 4a). According to the power−law relationship (current density ∝ *I_a_*^α^), an α value approaching unity indicates effective suppression of bimolecular recombination pathways [28,29]. Indeed, both OPD devices exhibit α > 0.98, confirming minimal bimolecular recombination losses. Figure 4b (V_OC_ vs. ln(*I_a_*)) yields the apparent ideality factors of 1.31 kT/q for PFBDT−8ttTPD:IT-4F and 1.52 kT/q for PFBDT−8ttTPD:Y6. An ideality factor (n) exceeding unity signifies trap−assisted recombination. Accordingly, the higher n value in the Y6−based devices compared to the IT−4F−based ones confirms more pronounced trap−mediated losses in the former. These recombination dynamic behaviors align with the GIWAXS observations, because the larger crystalline domains in the PFBDT−8ttTPD:IT−4F films reduce the trap site density in thick active layers.

Subsequently, energetic disorder in the photoactive layers was quantified using a sensitive EQE-based analysis. It involved determining the Urbach energy (E_U_), which characterizes the extent of band−tail disorder [34,35] and was extracted from the sub-bandgap slope of ln(EQE) vs. photon energy (*hν*) using the relation ln(EQE) = c + *hν*/Eᵤ [36]. As depicted in Figure 4c, the exponential rise of the absorption edge below the bandgap, commonly known as the Urbach tail, directly correlates with non−radiative recombination mediated by energetic disorder. The extracted E_U_ values are approximately 26.2 meV for PFBDT−8ttTPD:IT−4F and 31.4 meV for PFBDT−8ttTPD:Y6 devices. The reduced disorder in the PFBDT−8ttTPD:IT−4F blend is consistent with its larger coherence lengths, as observed using GIWAXS, suggesting more ordered and larger domains that effectively reduce trap-assisted recombination and contribute to the observed suppression of *J*_d_.

**Figure 5:**
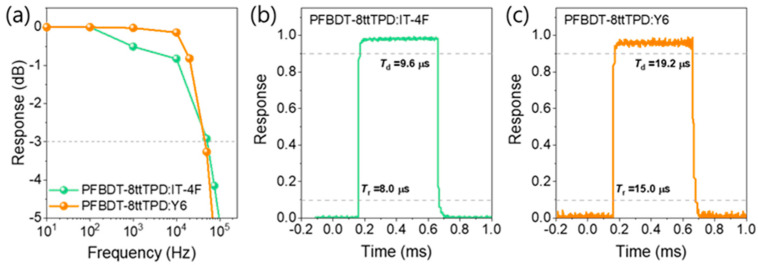
(**a**) Characterization of the dynamic behavior of both fabricated OPDs at −3dB cutoff frequencies. Transient photovoltage characteristics of (**b**) PFBDT−8ttTPD:IT−4F and (**c**) PFBDT−8ttTPD:Y6.

The linear dynamic range (LDR) is defined as 20log(*J*ₘₐₓ/*J*_min_) within the linear photocurrent regime, where *J*ₘₐₓ is the maximum *J*_ph_ and *J*_min_ the dark current density. LDR reaches 127 dB for PFBDT-8ttTPD:IT-4F under a reverse bias of −2.0 V, surpassing that of commercial silicon OPDs (120 dB) [37,38,39], and PFBDT-8ttTPD:Y6 also has a high LDR at ~112 dB. These exceptional LDR values directly reflect the pronounced trap-state suppression and reduced energetic disorder achieved via fluorination. The dynamic response was further characterized using the −3 dB cutoff frequency (f-3dB), the frequency at which the photocurrent responsivity falls to 1/√2 of its low-frequency value under the same bias. PFBDT-8ttTPD:IT-4F exhibits an f-3dB of 52.8 kHz, compared to 43.6 kHz for the Y6-based OPD (Figure 5a), and both rates meet the requirements for video−rate imaging and medical monitoring applications [40]. Transient photovoltage measurements under 0.5 s square−wave illumination (Figure 5b,c) yielded a rise time (*T_r_*, from 10% to 90%) of 8.0 µs and a fall time (*T*_f_, from 90% to 10%) of 10.1 µs for PFBDT-8ttTPD:IT-4F. In contrast, the Y6 devices exhibited comparatively longer response times (*T*_r_ ≈ 15.0 µs and *T*_f_ ≈ 19.2 µs) [41]. The relatively faster *T*_r_ of PFBDT-8ttTPD:IT-4F correlates with its faster charge−carrier mobility, whereas the shorter *T*_d_ arises from its lower dark-current level, together highlighting the advantages of fluorination for high-speed, low-noise detection using thick-film OPDs.

## 4. Conclusions

In conclusion, strategic fluorination in the PFBDT−8ttTPD backbone effectively overcomes thickness-induced performance losses in NFA-based OPDs. Our findings demonstrate that the thick active layers maintain ultra−smooth and pinhole-free morphologies, while GIWAXS analysis reveals enhanced π−π stacking and sustained long−range molecular order. The excellent charge-carrier mobilities inherent to the fluorinated polymer compensate for the weaker internal field in thicker films, thereby enabling efficient photocurrent extraction. Consequently, a drastic reduction in the dark current density is achieved, leading to outstanding detectivity and responsivity across the NIR spectrum. Thus, this fluorination approach enables the fabrication of high−performance OPDs with thick active layers via scalable solution processing, offering a promising route for robust imaging applications. Future studies will focus on molecular design strategies to broaden the spectral sensitivity and enhance the long-term operational stability.

## Figures and Tables

**Table 1:** Summary of the thickness-dependent performance for PFBDT−8ttPTD:NFAs under a reverse bias of –2.0 V.

	Thickness [nm]	*J*_d_ [A cm^−2^]	*J*_ph_ [A cm^−2^] ^c^	*R* [A W^−1^]	D* [Jones]
PFDBT−8ttTPD :IT−4F ^a^	250	8.74 × 10^−9^	2.18 × 10^−5^	0.43	2.52 × 10^12^
	350	7.43 × 10^−10^	2.00 × 10^−5^	0.43	2.77 × 10^13^
	450	4.08 × 10^−10^	2.06 × 10^−5^	0.43	3.78 × 10^13^
PFBDT−8ttTPD :Y6 ^b^	250	4.79 × 10^−8^	2.12 × 10^−5^	0.44	3.60 × 10^12^
	450	2.03 × 10^−9^	1.86 × 10^−5^	0.48	1.89 × 10^13^

^a^ Responsivity and detectivity measured for PFBDT-8ttTPD:IT-4F (@760 nm). ^b^ Responsivity and detectivity measured for PFBDT-8ttTPD:Y6 (@850 nm). ^c^ Photocurrent density measured under LED illumination (@810 nm, 55.66 µW·cm^−2^).

## Data Availability

The original contributions presented in this study are included in the article and Supplementary Material. Further inquiries can be directed to the corresponding authors.

## Supplementary Materials

The following supporting information can be downloaded at: https://www.mdpi.com/article/10.3390/polym17192663/s1, Scheme S1: The synthetic route of PFBDT−8ttTPD; Table S1: Comparison of the photodetection performance in the literature.
