# Supplementary material for: Unveiling the Role of Fluorination in Suppressing Dark Current and Enhancing Photocurrent to Enable Thick-Film Near-Infrared Organic Photodetectors
Source: Polymers (Basel). 2025 Oct 1;17(19):2663. doi: 10.3390/polym17192663 (PMC12526977; doi:10.3390/polym17192663)
Supplement: Supplementary file 1 [file polymers-17-02663-s001.zip › polymers-3884124-supplementary.pdf]

Supporting Information

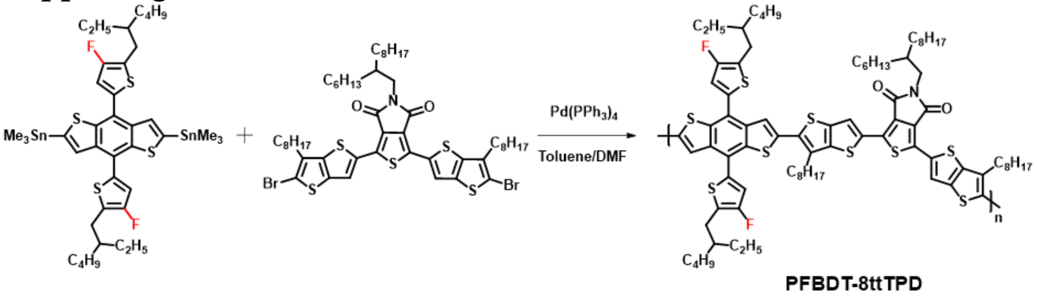

Scheme S1. The synthetic route of PFBDT-8ttTPD.

Table S1. Comparison of the photodetection performance in the literature.

	λ [nm]	R [A W^{-1}]	D^* [$\times 10^{13}$Jones]	Bias [V]	Thickness [nm]	Ref.
PFBDT-8ttTPD :IT-4F	760	0.43	3.78	-2.0	450	In this work
PFBDT-8ttTPD :Y6	850	0.48	1.89	-2.0	450	In this work
PTZBTTT-BDT :PCBM	800	0.10	1.75	-2.0	70	42
PFT-OEHp :Y6	800	0.50	1.16	-0.1	170	43
P3HT :P1	810	0.47	0.0000216	-1.0	230	44
PDPP-isoindigo1 :PCBM	824	0.159	1.40	-0.1	190	45
ANPHSO :PCBM	840	0.07	0.06	-1.0	-	46
PDTT(P4) :PCBM	850	0.061	0.14	-0.1	120	47
PDT :PCBM	900	0.037	0.19	-0.1	-	48
PNIR :ITIC	900	0.04	0.032	-0.5	-	49
PF :IT-4F	750	0.37	3.39	-0.5	100	50

42. X. Hu, Y. Dong, F. Huang, X. Gong, Y. Ca. Solution-Processed High-Detectivity Near-Infrared Polymer Photodetectors Fabricated by a Novel Low-Bandgap Semiconducting Polymer. J. Phy. Chem C, **2013**, 117, 6537.

43. Z. Zhong, F. Peng, Z. Huang, L. Ying, G. Yu, F. Huang, Y. Cao. High-Detectivity Non-Fullerene Organic Photodetectors Enabled by a Cross-Linkable Electron Blocking Layer. ACS Appl. Mater. Interfaces **2020**, 12 45092.

44. M. Casutt, M. Ruscello, N. Strobel, S. Koser, U. H. F. Bunz, D. Jänsch, J. Freudenberg, G. Hernandez-Sosa, K. Müllen. Diketopyrrolopyrrole-Polymer Meets Thiol–Ene Click Chemistry: A Cross-Linked Acceptor for Thermally Stable Near-Infrared Photodetectors. Chem. Mater. **2019**, 31, 7657.
45. J. Qi, J. Han, X. Zhou, D. Yang, J. Zhang, W. Qiao, D. Ma, Z.Y. Wang. Optimization of broad-response and high-detectivity polymer photodetectors by bandgap engineering of weak donor–strong acceptor polymers. Macromolecules, **2015**, 45, 3941.
46. G. M. Somashekharappa, C. Govind, V. Pulikodan, M. Paul, M. A. G. Namboothiry, S. Das, V. Karunakaran.
Unsymmetrical squaraine dye-based organic photodetector exhibiting enhanced near-infrared sensitivity J. Phys. Chem. C, **2020**, 124, 21730.
47. J. Qi, X. Zhou, D. Yang, W. Qiao, D. Ma, Z.Y. Wang. Optimization of solubility, film morphology and photodetector performance by molecular side-chain engineering of low-bandgap thienothiadiazole-based polymers. Adv. Funct. Mater., **2014** ,24, 7605.
48. J. Han, J. Qi, X. Zheng, Y. Wang, L. Hu, C. Guo, Y. Wang, Y. Li, D. Ma, W. Qiao, Z.Y. Wang. Low-bandgap donor–acceptor polymers for photodetectors with photoresponsivity from 300 nm to 1600 nm. J. Mater. Chem. C, **2017**, 5, 159.
49. H. J. Eun, H. Kye, D. Kim, I. S. Jin, J. W. Jung, S.-J. Ko, J. Heo, B.-G. Kim, and J. H. Kim. Effective Dark Current Suppression for High-Detectivity Organic Near-Infrared Photodetectors Using a Non-Fullerene Acceptor. ACS Appl. Mater. Interfaces, **2021**, 13, 11144.
50. U.-H. Lee, B. Park, S. Rhee, J.-W. Ha, D. R. Whang, H. J. Eun, J. H. Kim, Y. Shim, J. Heo, C. Lee, B. J. Kim, S. C. Yoon, J. Lee, S.-J. Ko. Achieving Highly Sensitive Near-Infrared Organic Photodetectors using Asymmetric Non-Fullerene Acceptor. Adv. Funct. Mater., **2020**, 19, 2185026
